# *Toxoplasma gondii* C2 Domain Protein Deletion Mutant as a Promising Vaccine Against Toxoplasmosis in Mice

**DOI:** 10.1111/1751-7915.70143

**Published:** 2025-05-23

**Authors:** Yifan Luo, Mingfeng He, Shengqiang Yang, Jiahui Qian, Zhengming He, Jiayin Xu, Liyu Guo, Siyu Xiao, Rui Fang

**Affiliations:** ^1^ State Key Laboratory of Agricultural Microbiology, College of Veterinary Medicine Huazhong Agricultural University Wuhan Hubei China; ^2^ College of Veterinary Medicine Huazhong Agricultural University Wuhan Hubei China

**Keywords:** C2 domain, immune protection, live attenuated vaccine, TGME49_203240, *Toxoplasma gondii*, vesicle transport

## Abstract

*Toxoplasma gondii* (*T. gondii*), a parasitic protozoan capable of infecting nearly all warm‐blooded animals, causes significant economic losses in livestock and poses a significant threat to both animal and public health. Despite its impact, no ideal vaccine is currently available to prevent toxoplasmosis. Vesicular transport plays a crucial role in the life cycle of *T. gondii*, and proteins involved in this process – such as those containing C2 domains – may serve as novel targets for the development of live attenuated vaccines. In this study, we evaluated the feasibility of a C2 domain‐containing protein (TGME49_203240) as a live attenuated vaccine candidate. Our findings suggest that TGME49_203240 may be involved in vesicular transport and that it is essential for *T. gondii* growth. Deletion of TGME49_203240 reduced parasite virulence and impaired tissue cyst formation in mice. Moreover, mice vaccinated with ME49*Δ203240* were protected against the lethal challenge of the tachyzoites of *T. gondii* I, II, III strains and cysts of II strain. In addition, the ME49*Δ203240* strain elicited robust immune responses, including the production of high levels of specific IgG antibodies and key cytokines (IFN‐γ, TNF‐α and IL‐12). These findings highlight TGME49_203240 as a promising target for the development of a live attenuated vaccine against *T. gondii*.

## Graphical abstract

Without TGME49_203240, a C2 domain‐containing protein, the virulence of *T. gondii* is much weakened. Immunisation of mice with the ME49Δ203240 strain produces a Th1‐biased immune response against reinfection by the *T. gondii* I, II and III strains.

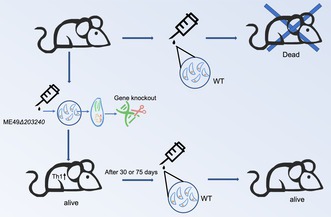

## 1 Introduction

*Toxoplasma gondii* is a highly specialised intracellular parasite that causes zoonotic infections in nearly all warm‐blooded animals and has a widespread global prevalence (Rostami et al. 2020). Its life cycle is notably complex, with sexual reproduction occurring exclusively in felines, the definitive hosts, while asexual replication takes place in intermediate hosts. Toxoplasmosis can be a life‐threatening disease, particularly for immunocompromised individuals, such as those with AIDS or cancers. If primary infection occurs during pregnancy, it can lead to miscarriage, stillbirth, congenital toxoplasmosis, fetal deformities and other severe complications (Nayeri et al. 2020; Almeria and Dubey 2021). Beyond its impact on human health, toxoplasmosis also causes substantial economic losses in livestock industries, particularly in small ruminants and swine production, leading to abortions, reduced animal product quality and significant zoonotic risks (Foroutan et al. 2019; Nayeri et al. 2021; Su et al. 2022). The development of a *T. gondii* vaccine remains a global challenge. Currently, only one commercial vaccine (Toxovax) is produced by attenuating T. *gondii* through multiple passages after isolating it from lambs. However, Toxovax is only approved for use in sheep, with a high risk of reversion to its virulence (Buxton and Innes 1995). Therefore, there is an urgent need to develop safe and effective vaccines as well as therapeutic strategies for the prevention, control and treatment of *Toxoplasma* infections.

Currently, multiple *T. gondii* vaccine candidates have been developed. However, inactivated vaccines have proven ineffective in eliciting robust immunoprotection in animals (Stanley et al. 2004; Saavedra et al. 2004). While subunit vaccines and DNA vaccines have demonstrated only partial immunoprotection, live attenuated vaccines have exhibited the strongest immunoprotective efficacy. As a result, live attenuated vaccines are considered the most promising strategy for *T. gondii* vaccine development (Bhopale 2003; Dunay et al. 2018; Onile et al. 2020; Chu and Quan 2021). With the advancement in gene editing technologies, the development of genetically modified live attenuated vaccines has become a major research focus. Techniques such as CRISPR/Cas9 and Cre‐loxp have enabled the precise deletion of virulence‐related genes, paving the way for safer and more effective vaccine strains. Several gene‐deletion‐based live vaccine strains, including *ΔADSL*, *Δα‐AMY* and *ΔOMPDCΔUP*, have been developed. However, their safety and long‐term efficacy still require further validation (Fox and Bzik 2010, 2015; Wang et al. 2020; Lyu et al. 2021).

In the field of live attenuated vaccines, most vaccines have been designed by targeting genes associated with parasite adhesion, invasion, replication and biosynthetic pathways of parasites (Fereig and Nishikawa 2016; Chu and Quan 2021). For example, microneme protein (MIC) is a class of proteins released by *T. gondii* during cell invasion. Low‐virulence RH strains with MIC1 and MIC3 genes knockout have been shown to protect mice against *T. gondii* cysts challenge (Cerede et al. 2005). Another promising approach involves disrupting the de novo uracil synthesis pathway. Deletion of two enzymes critical – the uridine phosphorylase (UP) and orotidine‐5′‐monophosphate decarboxylase (OMPDC) – renders *T. gondii* incapable of growth in standard environments, requiring high concentrations of uracil to persist. Mice immunised with either the UP or OMPDC deficient strains exhibited resistance to the challenge of *T. gondii* type I strain (Fox and Bzik 2010, 2015; Wilson et al. 2012). Similarly, Adenylosuccinate lyase (ADSL), an enzyme involved in the adenylate synthesis pathway, has been identified as a potential target for attenuation. Mice immunised with ADSL‐deficient *T. gondii* strains developed high levels of specific antibodies, leading to a significant reduction in tissue cyst formation (Wang et al. 2020).

Vesicular transport plays a crucial role in various biological processes and disrupting this pathway can significantly impact the growth and virulence of *T. gondii*. Therefore, proteins involved in vesicular transport may serve as potential antiparasitic targets. Proteins containing the C2 domain were speculated to be involved in the process of vesicular transport, as the C2 domain can bind a variety of substances, including phospholipids, inositol polyphosphates and other substances involved in membrane translocation and signal transduction (Cho and Stahelin 2006; Zhang and Aravind 2010; Larsen and Sansom 2021; Jahn et al. 2024). Given their functional significance, the C2 protein family may serve as a potential target for the development of live attenuated *T. gondii* vaccines.

In this study, we identified TGME49_203240, a C2 domain‐containing protein that partially co‐localised with the endoplasmic reticulum. Deletion of the TGME49_203240 gene significantly impacted the growth and virulence of *T. gondii*. We further evaluated the potential of the TGME49_203240 gene knockout strain (ME49*Δ203240*) as a live attenuated vaccine and found that ME49*Δ203240* provided potent immunoprotection against tachyzoite challenge from *T. gondii* types I, II and III, as well as cyst challenge from the type II strain in mice. Moreover, ME49*Δ203240* induced a robust humoral immune response and a Th1‐biased cellular immune response. These findings highlight TGME49_203240 as a promising target for developing a live attenuated *T. gondii* vaccine, offering a novel approach to toxoplasmosis prevention.

## 2 Experimental Procedures

### 2.1 Parasite Strains and Cell Lines

RH*Δku80*, RH*ΔHXGPRT*, ME49 and VEG were kept in our laboratory. 203240‐3HA (TGGT1_203240 localised strain), ME49*Δ203240* (TGME49_203240 gene knockout strain) and Comp‐*Tg*203240 (ME49*Δ203240* complementation strain) strains were constructed in this study and propagated in human foreskin fibroblasts (HFF).

Human foreskin fibroblasts (HFF) were cultured in a DMEM medium containing 10% fetal bovine serum (FBS, HY, China) and 1% penicillin–streptomycin (Beyotime, China) at a ratio of 3:1 for cell passage.

*T. gondii* tachyzoites were propagated in HFF cells in a DMEM medium containing 2% FBS and 1% penicillin–streptomycin.

All cells and parasites were cultured at 37°C, 5% CO_2_.

### 2.2 Bioinformatics Analysis

The sequence of TGME49_203240 was obtained from the ToxoDB database and subjected to analysis in the InterPro database. The conserved sequence of the protein structural domain of the C2D_Tricalbin‐like protein family was downloaded from the NCBI database. The protein sequences were imported into DNASTAR 11 Protean for analysis of antigenic epitopes and hydrophobicity.

### 2.3 Construction of TGME49_203240 Gene Knockout Strain and Complementation Strain

The corresponding gRNAs were designed using the ToxoDB database, and the CRISPR plasmids were constructed by replacing the UPRT target RNA (gRNA) in pSAG1‐Cas9‐sgUPRT with the corresponding gRNA by Q5 targeted mutagenesis kit (NEB, USA). Homology templates used for gene replacements (pTGME49_203240::DHFR) were constructed by multi‐fragment ligation using the ClonExpress II one‐step cloning kit (Vazyme, Nanjing, China). Briefly, the 5′ and 3′ homology arms of the gene were amplified from the genomic DNA of the ME49 strain and cloned into the pUC19 vector along with DHFR. The CRISPR plasmid targeting TGME49_203240 and the homologous templates, which displace TGME49_203240, were cotransfected into fresh ME49 tachyzoites (Shen and Sibley 2014; Yang et al. 2017). The correct positive clone (ME49*Δ203240*) was screened by the pyrimethamine and identified by PCR (Shen and Sibley 2014; Yang et al. 2017). To construct the complementation strain, we replaced TgLDH 1 in pCom‐LDH 1 (Xia et al. 2018) with the CDS sequence of TGME49_203240. Then pTub::TGME49_203240‐CAT fragment was transfected into ME49*Δ203240*, and the correct complementation strain (Comp‐*Tg*203240) was identified through the use of a drug (chloramphenicol), a limited dilution method, PCR and IFA. All primer sequences are shown in Table 1. The Western Blot and IFA were performed as described previously (Sugi et al. 2017).

**TABLE 1:** Primers used in this study.

Primer	Sequence 5′‐3′	Use
gRNA‐R	AACTTGACATCCCCATTTAC	To construct gene‐specific CRISPR plasmids
gRNA‐203240‐3HA‐F	GCATGACAGGGAACGCCATGGTTTTAGAGCTAGAAATAGC	To construct the 203240‐3HA specific CRISPR plasmid
5H‐3HA‐DHFR‐F	GGCGCACGCGGCAGAACCTCGTAGCTTCACCCTTCATCCCTCGAGCAGCTCGCTACCCCTACGATGTGCCGGATT	Amplification of homologous templates 5H‐3HA‐DHFR‐3H for203240‐3HA strain construction
3H‐3HA‐DHFR‐R	CTTTCCTGACCAACAGTCGCCCCCCTCTCTTCCCGCTCCTCTGTGTCGGCCACCGCTTTCTCAACAGGAAAAAGC	Amplification of homologous templates 5H‐3HA‐DHFR‐3H for203240‐3HA strain construction
203240‐3HA‐PCR1‐F	GCGAGTGTGACTATGTGGCG	PCR1 of 203240‐3HA
203240‐3HA‐PCR1‐R	CGTACTATGGCCGGTCGATG	PCR1 of 203240‐3HA
203240‐3HA‐PCR2‐F	CCGCGAACGATTGGCGATACGTC	PCR2 of 203240‐3HA
203240‐3HA‐PCR2‐R	GCTCGCACTCACCACACAGC	PCR2 of 203240‐3HA
gRNA‐ME49*Δ*203240‐F	AGACGCCATACCCCGCACAGGTTTTAGAGCTAGAAATAGC	To construct the ME49*Δ203240* specific CRISPR plasmid
ME49*Δ203240*‐5H‐F	GTTGTAAAACGACGGCCAGTAATGTTTCTGCGTCCCCTGT	Amplification of 5′‐homology of TGME49_203240 for p203240:: DHFR homologous recombinant plasmid construction
ME49*Δ203240*‐5H‐R	GATGTCTTCTGCGCGGGTTGTTTTTAGACAAGAAAGGCCT	Amplification of 5′‐homology of TGME49_203240 for p203240:: DHFR homologous recombinant plasmid construction
ME49*Δ203240*‐3H‐F	GCCACAAGTTCAGCGTGTCCGGCTCCTACGGGGTTTCCTC	Amplification of 3′‐homology of TGME49_203240 for p203240:: DHFR homologous recombinant plasmid construction
ME49*Δ203240*‐3H‐R	GCTATGACCATGATTACGCCACCGCTCGCACTCACCATAC	Amplification of 3′‐homology of TGME49_203240 for p203240:: DHFR homologous recombinant plasmid construction
PUC19‐F	GGCGTAATCATGGTCATAGCTGT	Amplification of pUC19 vector for p203240:: DHFR homologous recombinant plasmid construction
PUC19‐R	ACTGGCCGTCGTTTTACAACG	Amplification of pUC19 vector for p203240:: DHFR homologous recombinant plasmid construction
DHFR‐F	CAACCCGCGCAGAAGACATC	Amplification of DHFR for p203240:: DHFR homologous recombinant plasmid construction
DHFR‐R	GGACACGCTGAACTTGTGGC	Amplification of DHFR for p203240:: DHFR homologous recombinant plasmid construction
ME49*Δ203240*‐PCR1‐F	GTTCTCTCGCGAGGCACTTT	PCR1 of ME49*Δ203240*
ME49*Δ203240*‐PCR1‐R	GATTTGTGAGGACGACTCAC	PCR1 of ME49*Δ203240*
ME49*Δ203240*‐PCR2‐F	CACGACAGCAGACAACTTTC	PCR2 of ME49*Δ203240*
ME49*Δ203240*‐PCR2‐R	CACTTGCGGTTCTTGTGACC	PCR2 of ME49*Δ203240*
ME49*Δ203240*‐PCR3‐F	GTCGGTGAACTCTCTTGTGC	PCR3 of ME49*Δ203240*
ME49*Δ203240*‐PCR3‐R	TCAGGGGAGGAAGATAAGCC	PCR3 of ME49*Δ203240*
gRNA‐Comp‐F	CCTCGCCGAAGTAAGTTCTGGTTTTAGAGCTAGAAATAGC	To construct gene specific CRISPR plasmids
Comp‐*Tg*203240‐PCR4‐F	CGGTTGGTGATCCTGGTTGGAC	PCR4 of Comp‐*Tg*203240
Comp‐*Tg*203240‐PCR4‐R	CTGAGACCTGCGGTGCAGAAG	PCR4 of Comp‐*Tg*203240
Comp‐*Tg*203240‐PCR5‐F	GCATGTTCGGTTGTACATCG	PCR5 of Comp‐*Tg*203240
Comp‐*Tg*203240‐PCR5‐R	GCGAATACACATAGTCACACCG	PCR5 of Comp‐*Tg*203240
TGME49_203240‐CDS‐F	CTTACGATGTTCCAGATTATGCCGCACCTGCTGCTCCGAACAAG	Amplification of TGME49_203240‐CDS::HA for pTub::TGME49_203240::HA::CAT construction
TGME49_203240‐CDS‐R	GCAGCTTCTGTGGGCTGCATAGCTGCTCGAGGGATGAAGGGTGAAG	Amplification of TGME49_203240‐CDS::HA for pTub:: TGME49_203240::HA::CAT construction
Comp‐TGME49_203240‐F	CATGATTACGCCAAGCTCGG	Amplification of TGME49_203240‐CDS::HA for pTub:: TGME49_203240::HA::CAT construction
Comp‐TGME49_203240‐R	AAACGACGGCCAGTGAATTG	Amplification of TGME49_203240‐CDS::HA for pTub:: TGME49_203240::HA::CAT construction
pET32a‐F	GTCGACAAGCTTGCGGCCGCACTCG	Amplification of pET32a vector for all the *E. coli* transfection plasmid construction
pET32a‐R	ATGATGATGATGATGGTGCATATGGCC	Amplification of pET32a vector for all the *E. coli* transfection plasmid construction
TGME49_203240‐CDS‐F	CTTACGATGTTCCAGATTATGCCGCACCTGCTGCTCCGAACAAG	Amplification of TGME49_203240‐CDS for pET32a‐TGME49_203240 construction
TGME49_203240‐CDS‐R	GCAGCTTCTGTGGGCTGCATAGCTGCTCGAGGGATGAAGGGTGAAG	Amplification of TGME49_203240‐CDS for pET32a‐TGME49_203240 construction
pET32a‐TGME49_203240‐F	ATGCACCATCATCATCATCATGCACCTGCTGCTCCGAACAA	Construction of pET32a‐TGME49_203240 plasmid for *E. coli* protein expression
pET32a‐TGME49_203240‐R	GCGGCCGCAAGCTTGTCGACAGAGTAGCCCTGGGGCAGGA	Construction of pET32a‐TGME49_203240 plasmid for *E. coli* protein expression
RT‐tubulin‐F	CACTGGTACACGGGTGAAGGT	For qPCR detection in parasite burden assay
RT‐tubulin‐R	ATTCTCCCTCTTCCTCTGCG	For qPCR detection in parasite burden assay

### 2.4 Localisation Assay of TGME49_203240

The 203240‐3HA strain was constructed by transfecting the targeting CRISPR plasmid and a homologous template with a 3HA‐DHFR tag into fresh RHΔ*ku80* tachyzoites by CRISPR/Cas9 gene editing. It was also characterised by screening with 1 μM pyrimethamine, PCR and IFA.

Fresh tachyzoites of the 203240‐3HA strain were transferred to glass slides full of HFF cells for overnight growth and then fixed with 4% paraformaldehyde at room temperature, permeabilised with 0.1% Triton X‐100, closed in 10% FBS (diluted with PBS) at 37°C and stained with mouse‐anti‐HA, mouse‐anti‐GRASP, mouse‐anti‐GAP50, mouse‐anti‐MIC‐2, rabbit‐anti‐*Tg*ALD, rabbit‐anti‐*Tg*SERCA, rabbit‐anti‐GRA7, rabbit‐anti‐ROP17 and rabbit‐anti *Tg*GAP45 as primary antibodies. Alexa‐488‐R, Alexa‐596‐M and Hoechst were used as secondary antibodies for staining, and after adding an anti‐fluorescence quencher, the staining was observed under a fluorescence microscope.

### 2.5 Preparation of Polyclonal Antibodies to TGME49_203240

The CDS sequence of TGME49_203240 was amplified from the cDNA of the ME49 strain. Following this, it was ligated with the pET32a vector to construct the pET32a‐203240 prokaryotic expression plasmid. Protein expression was then carried out with *E. coli* BL21(DE3) engineering bacteria. Once the bacterial culture had reached the logarithmic growth phase (as indicated by an OD600 reading of 0.5–0.6), the protein expression process was initiated by the addition of IPTG (1 mM) and continued for 4 h at 37°C. Following the disruption of the bacterial fluid, inclusion body proteins were isolated, denatured and purified using a nickel column. Finally, the purified proteins were identified through SDS‐PAGE analysis.

The purified proteins were combined with the Fourier adjuvant in a 1:1 ratio and subsequently administered to mice via subcutaneous injection at separate points. Each mouse received 75 μg of protein. The initial immunisation was conducted with complete F‐adjuvant, while all subsequent immunisations were performed with incomplete F‐adjuvant. Following the fourth immunisation, serum was collected. The binding capacity of the antigen and polyclonal antibody was evaluated through Western blot analysis. (All the results are in the Supplementary chart Figure S1).

### 2.6 Plaque Assay

The fresh tachyzoites of *T*. *gondii* were inoculated into 6‐well plates full of HFF cells (200 *Tg*/per well) and cultured for 10 days at 37°C in a 5% CO_2_ incubator. After the medium was washed away with PBS, the infected HFF cells were fixed with 4% paraformaldehyde at room temperature, stained with 0.1% crystalline violet at 37°C, dried and the pictures were scanned with a scanner.

### 2.7 Invasive Capability Assay

Purified tachyzoites were inoculated onto glass slides pre‐seeded with HFF cells (10^6 *Tg*/well) and incubated at 37°C for 15 min. After washing three times with PBS, the cells were fixed with 4% paraformaldehyde at room temperature for 7 min. Subsequently, the slides were incubated with mouse anti‐*T. gondii* IgG antibody (1:100 dilution) at 37°C for 30 min, followed by permeabilisation with 0.1% Triton X‐100 for 20 min at 37°C and blocking nonspecific binding with 10% FBS (diluted in PBS) at 37°C for 20 min. Next, the slides were incubated with rabbit anti‐*Tg*ALD antibody at 37°C for 30 min, followed by staining with Alexa‐488‐R, Alexa‐596‐M and Hoechst for 20 min. The number of invasive and non‐invasive *T. gondii* tachyzoites and host cell nuclei were quantified under a fluorescence microscope. A total of 15–20 fields of view were analysed, with at least 200 tachyzoites counted per experiment. The invasion rate was calculated as follows: (number of invaded *T. gondii*—number of uninvaded *T. gondii*) / total number of nuclei.

### 2.8 Replication Assay

Freshly purified *T*. *gondii* tachyzoites were inoculated into the glass slides full of HFF cells at 37°C for 1 h. Uninvaded *T*. *gondii* tachyzoites were washed away with PBS, and incubation was continued at 37°C for 24 h. Then, the cells were treated as above. The number of parasites per parasitophorous vacuole (PV) was calculated, and at least 200 PVs were counted in each group.

### 2.9 Parasite Burden Assay

Seven‐week‐old female ICR mice were injected intraperitoneally with 1 × 10^4^ ME49*Δ203240* or wild‐type ME49 tachyzoites, respectively, in 5 mice per group. After 5 days of infection, the mice were euthanised, and the peritoneal fluids were collected and centrifuged. Then, the gDNA was extracted by taking 1/20 of the mixed ascites for the detection of the parasite‐loaded volume using qPCR.

### 2.10 Detection of *T. gondii* Virulence and Tissue Cyst‐Forming Ability

Purified fresh *T. gondii* tachyzoites were counted and diluted with FBS‐free DMEM medium. For the virulence experiments, each mouse was injected with 200 ME49*Δ203240* tachyzoites, and the control was injected with the same number of wild‐type ME49 strains. Brain tissues of mice were collected after infected parasites for 30 days, and the number of tissue cysts was detected by DBA‐FITC.

For the gradient virulence experiments, each mouse was injected with 10^2^, 10^3^, 10^4^ and 10^5^ ME49*Δ203240* strains, and the control group was injected with 200 tachyzoites of wild‐type ME49 strains.

### 2.11 HE Staining of the Liver, Spleen and Lung

After mice were immunised with 200 ME49*Δ203240* and ME49 tachyzoites for 30 days, the liver, lungs, spleen and liver were taken and fixed in 4% paraformaldehyde at room temperature for 24 h (3 mice per group), dehydrated with ethanol, cleared with xylene, embedded in wax and sliced with a slicer. The unimmunised mice were used as controls. Sections were then stained with haematoxylin and eosin (H&E).

### 2.12 Immunoprotection Against Acute *T. gondii* and Chronic Infection

Female ICR mice were immunised with 200 tachyzoites of ME49 *Δ203240* strains through intraperitoneal injection at 30‐ or 75‐day post‐immunisation. Then, the immunised mice were challenged with 10^4^ tachyzoites of RH*ΔHXGPRT*, ME49, or VEG, by intraperitoneal injection (10 mice for each group), or 50 fresh cysts of ME49 by oral administration. The survival curves of the mice were observed, recorded and statistically analysed for 30 days.

### 2.13 Detection of Serum *T. gondii*‐Specific Antibodies and Cytokines by ELISA

Sera from mice immunised with the 200 ME49*Δ203240* strain for 30, 75 and 125 days were collected. The TSA of *T. gondii* is prepared as in this description (Yang et al. 2020). The 96‐well plates were coated overnight with TSA at a concentration of 16 μg/mL. After blocking, mouse serum (diluted 1:100) was added and incubated at 37°C for 45 min. Subsequently, the plates were incubated with goat anti‐mouse IgG‐HRP, goat anti‐mouse IgG2a and goat anti‐mouse IgG1 for 30 min. Colour development was initiated by adding TMB substrate for 5 min, followed by the addition of a stop solution. The OD630 was measured using a microplate reader.

Cytokines were detected by the Elisa Max Deluxe Set Mouse kit (Biolegend, USA) for IL‐4, IL‐12, IFN‐γ and TNF‐α.

### 2.14 Statistical Analysis

Statistical analyses were performed in Prism 10 software (GraphPad Software Inc., La Jolla, CA, USA) with unpaired *t*‐test, two‐way ANOVA test, Mantel‐Cox log‐rank test, *p* > 0.05 for ns (not significant differences), *p* < 0.05 for * (statistical difference), *p* < 0.01 for ** (statistically significant difference) and *p* < 0.001 for *** (extremely statistically significant difference).

## 3 Results

### 3.1 Bioinformatics Analysis

**FIGURE 1:**
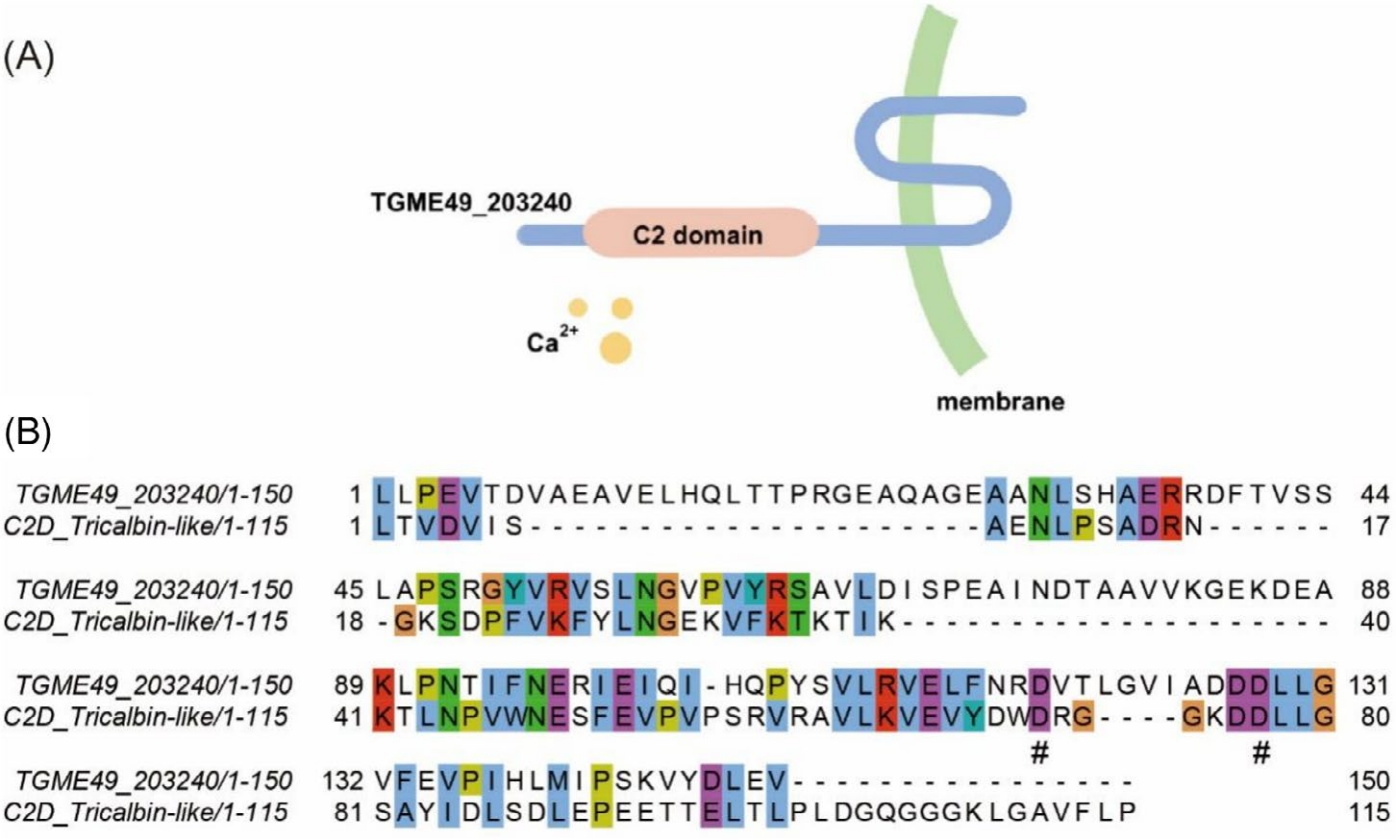
Bioinformatics analysis of TGME49_203240. (A) Schematic structure of TGME49_203240. Data from InterPro. (B) Comparison of the structural domain of the C2 domain of TGME49_203240 with conserved sequences of the C2D_Tricalbin‐like family. Putative Ca^2+^ ion‐binding sites are labelled with the ‘#’ under the sequence.

Using the ToxoDB database and InterPro database, we identified TGME49_203240, a protein containing C2 domains which may be involved in vesicle transport in *T. gondii*. It has a C2 domain at the N‐terminal (amino acids 100–249) and three transmembrane structural domains at the C‐terminal end (Figure 1A). However, not all C2 domains are functionally active; some serve merely as structural scaffolds or remain nonfunctional (Cho and Stahelin 2006; Zhang and Aravind 2010; Corbalan‐Garcia and Gomez‐Fernandez 2014; Larsen and Sansom 2021). To further investigate TGME49_203240, we conducted a sequence comparison of its C2 domain using the NCBI database. Our results revealed that the sequence of the TGME49_203240 C2 domain protein exhibits a high degree of similarity to the sequence of the C2 domain protein family of C2D_Tricalbin‐like proteins which are involved in membrane transport and sorting, suggesting that the C2 domains of TGME49_203240 may be functionally active (Figure 1B) (Essen et al. 1996; Xu et al. 1998; Verdaguer et al. 1999). Based on these findings, we hypothesise that TGME49_203240 may be involved in the process of vesicle transport in *T. gondii*.

### 3.2 TGME49_203240 Is Partially Co‐Localised With *T. gondii* Endoplasmic Reticulum

**FIGURE 2:**
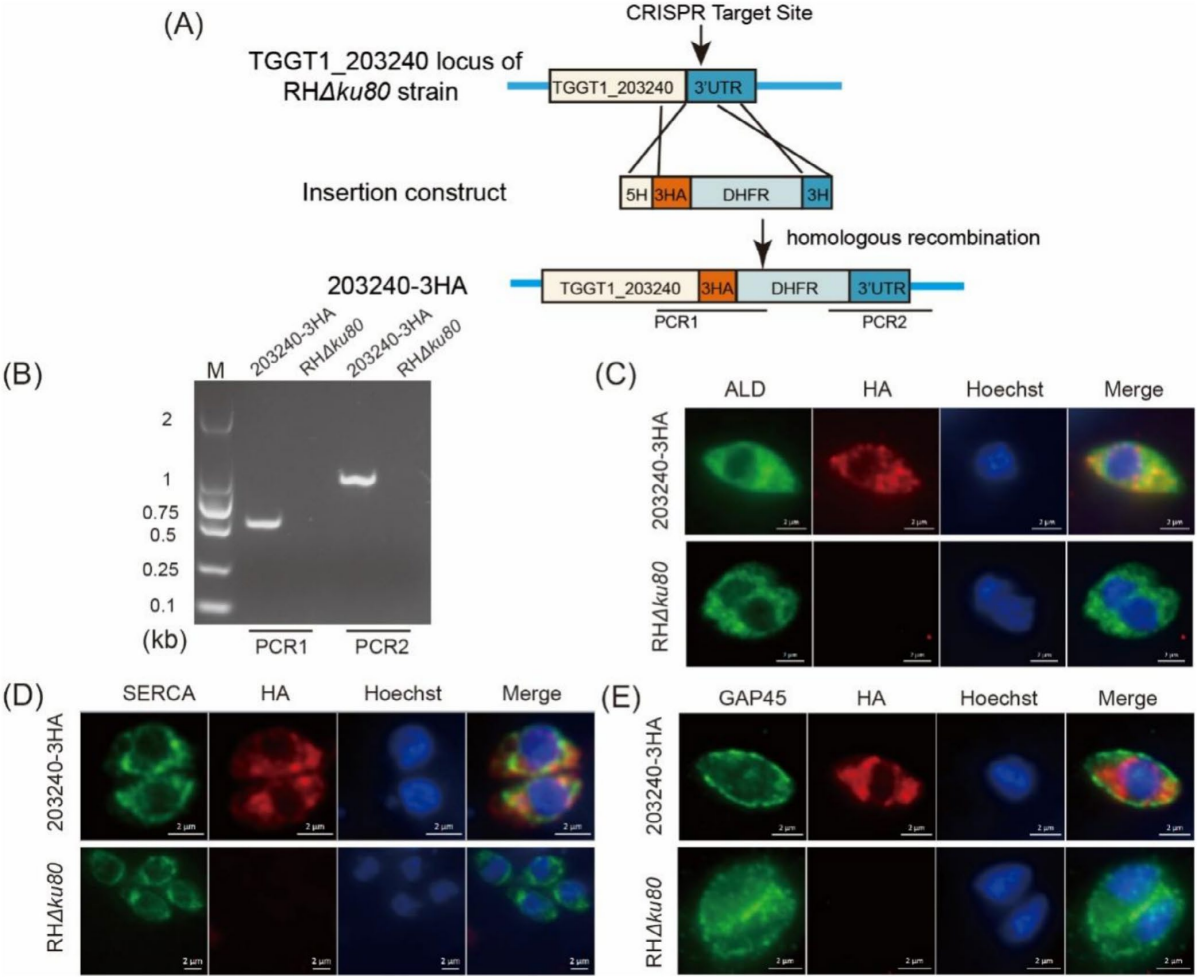
Localisation assay of TGME49_203240. (A) Schematic diagram of 203240‐3HA strain construction. The 3HA‐DHFR tag was integrated into the TGGT1_203240 gene of RH*Δku80* strain by CRISPR/Cas9 gene editing system. (B) PCR identification of 203240‐3HA strain. PCR1 and PCR2 represent the correct position of the 3HA‐DHFR insertion into the RH*Δku80* strain. (C–E) IFA to determine TGME49_203240 subcellular localisation. Scale bar = 2 μm. (C) Immunofluorescence analysis of the transgenic parasites with mouse anti‐HA and rabbit anti‐*Tg*ALD antibodies. (D) Immunofluorescence analysis of the transgenic parasites with mouse anti‐HA and rabbit anti‐*Tg*SERCA antibodies. (E) Immunofluorescence analysis of the transgenic parasites with mouse anti‐HA and rabbit anti‐*Tg*GAP45 antibodies.

To further explore whether TGME49_203240 is involved in the vesicular transport process of *T*. *gondii*, its subcellular localisation needs to be determined. Thus, the 203240‐3HA strain was constructed, and the PCR identification confirmed its successful generation (Figure 2A,B). Subsequently, the subcellular localisation of TGME49_203240 was determined by indirect immunofluorescence (IFA) (Figure 2C–E). The results showed that the HA tag was partially co‐localised with cytoplasmic protein (*Tg*ALD) and endoplasmic reticulum (*Tg*SERCA). However, it does not colocalise with gliding‐associated protein (*Tg*GAP45). These findings suggest that TGME49_203240 primarily functions in the endoplasmic reticulum and cytoplasm of *T. gondii*. Additionally, a murine polyclonal antibody against TGME49_203240 was generated to examine potential differences in localisation across different *T. gondii* strains. The results indicated that TGME49_203240 localisation remains consistent between RH and ME49 strains (Figure S2).

### 3.3 TGME49_203240 Is Important for *T. gondii* Growth In Vitro

**FIGURE 3:**
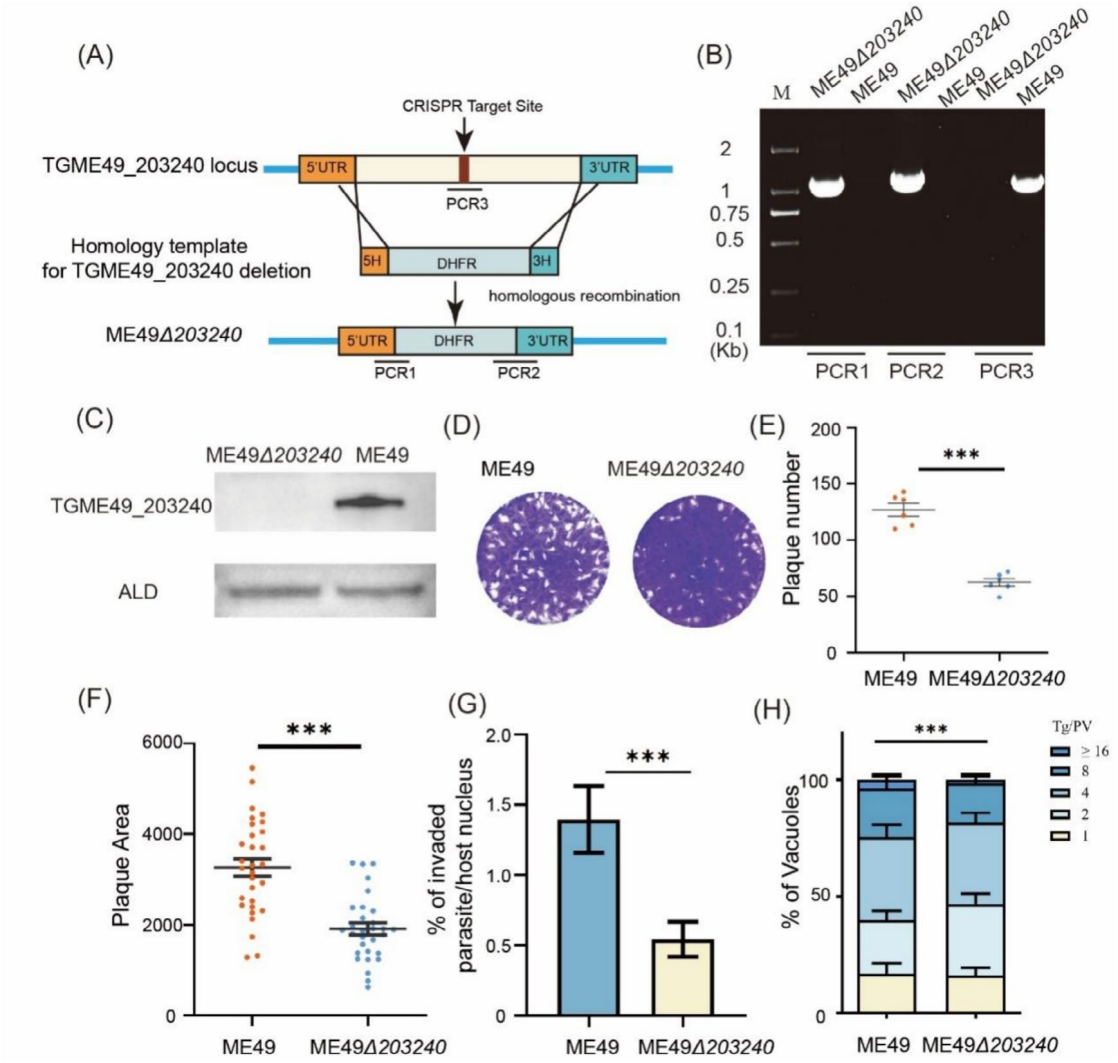
Deletion of TGME49_203240 and effects on growth of *T. gondii* in vitro. (A) Schematic diagram of TGME49_203240 gene deletion strain construction. (B) PCR identification of ME49*Δ203240* strain with deletion of TGME49_203240. PCR1 and PCR2 represent the successful insertion of the DHFR tag into the correct gene position, and PCR3 represents the successful deletion of the TGME49_203240 gene. (C) Western Blot identification of ME49*Δ203240* strain with deletion of TGME49_203240. Mouse‐anti‐TGME49_203240 and rabbit‐anti‐*Tg*ALD as primary antibodies. (D) Plaque assay of ME49*Δ203240* and ME49 strain. (E) Plaque number of ME49 and ME49*Δ203240* strain, ***p < 0.001, unpaired *t*‐test. (F) Plaque area of ME49 and ME49*Δ203240* strain, ***p < 0.001, unpaired *t*‐test. (G and H) In vitro growth assay of strain ME49 and ME49*Δ203240* strain with mouse‐anti‐*T. gondii* and rabbit‐anti‐*Tg*ALD. (G) Invasion assay of ME49 and ME49*Δ203240* strain, ***p < 0.001, two‐way ANOVA. (H) Replication assay of ME49*Δ203240* and ME49 strain, ***p < 0.001, two‐way ANOVA.

To investigate the role of TGME49_203240 in *T. gondii*, a knockout strain (ME49*Δ203240*) was generated using CRISPR/Cas9 gene‐editing technology (Figure 3A). Following pyrimethamine selection, the correct mutant strain was identified by PCR analysis (Figure 3B). Western blot analysis further confirmed the successful deletion of TGME49_203240 at the protein level (Figure 3C).

To assess the impact of TGME49_203240 gene deletion on the growth of *Toxoplasma* tachyzoites in vitro, the phenotype assays of ME49*Δ203240* were performed. Plaque assay showed that the size and number of plaques formed by strain ME49*Δ203240* were significantly different from those formed by wild‐type strain ME49, with plaques formed by strain ME49*Δ203240* being smaller than those formed by wild‐type strain ME49, and with fewer plaques formed by strain ME49*Δ203240* (Figure 3D–F). Additionally, the results of the invasion capability assay showed that the deletion of the TGME49_203240 gene significantly reduced the invasion ability of the ME49*Δ203240* strain compared with the ME49 strain (Figure 3G). Besides, the replication assay demonstrated that the ME49*Δ203240* strain exhibited significantly slower replication in vitro compared to the wild‐type ME49 strain. Collectively, these results demonstrated that TGME49_203240 gene deletion significantly impairs the in vitro growth of *Toxoplasma* tachyzoites (Figure 3H).

Furthermore, we aimed to investigate whether the deletion of TGME49_203240 affects the localisation of several organelle markers involved in *T. gondii* vesicle transport. Notably, the deletion of TGME49_203240 did not significantly alter the localisation of cytoplasmic, Golgi, rhoptry, microneme, dense granule, or gliding‐associated proteins (Figure S3).

### 3.4 TGME49_203240 Gene Deletion Affects the Proliferation Rate of *T. gondii* in Mice

**FIGURE 4:**
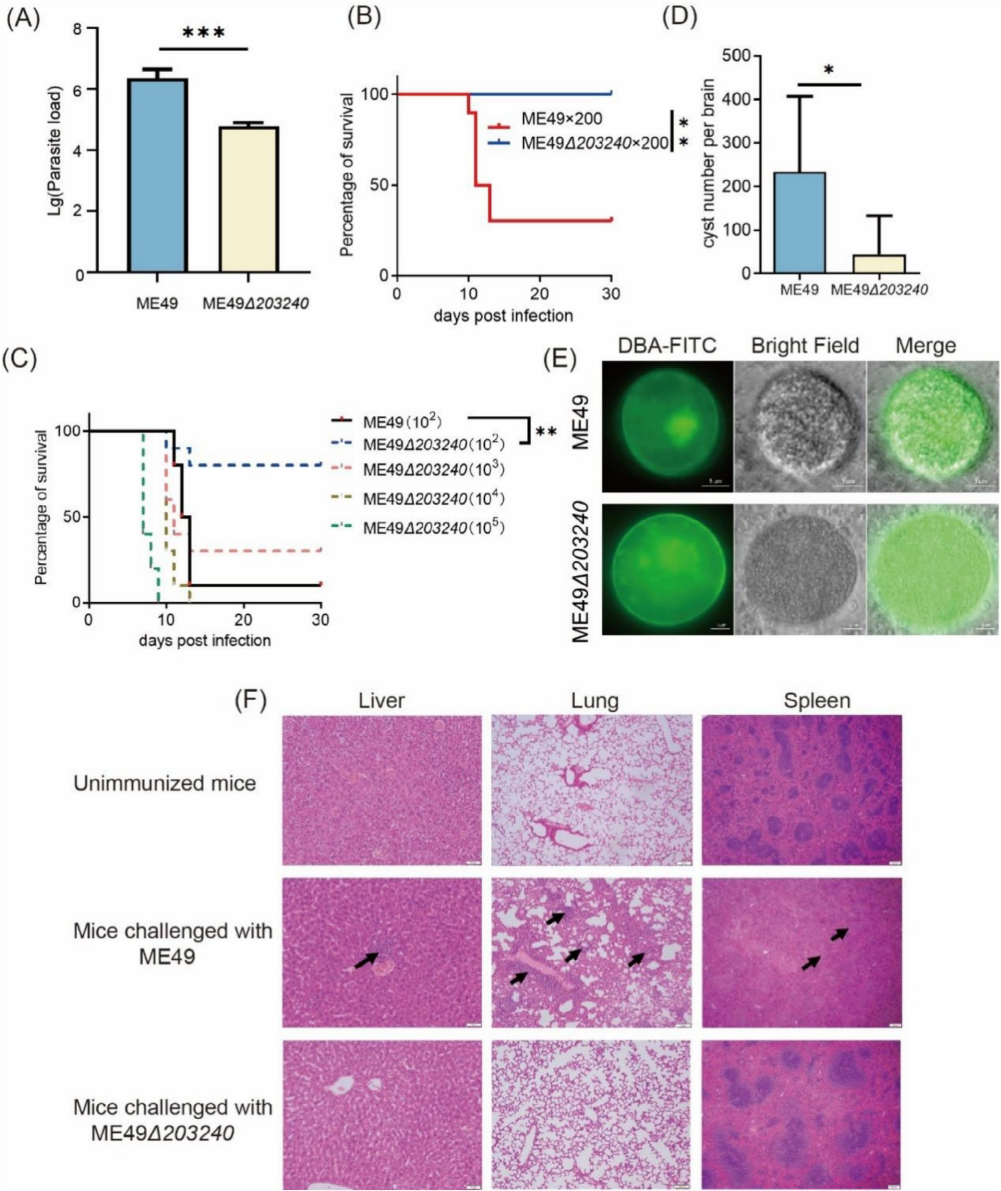
TGME49_203240 deletion results in reduced parasite virulence and reduced tissue cyst formation. (A) Parasite load experiment of ME49 and ME49*Δ203240* in mouse. 7‐week‐old female ICR mice were injected with 10^4^ ME49 or ME49*Δ203240* strain, and parasite burdens in the peritoneal fluid of mice were detected after infecting 5 days by qPCR, ***p < 0.001, unpaired *t*‐test. (B) Virulence test, 7‐week‐old ICR mice, 10 in each group, were infected with 200 ME49 or ME49*Δ203240* strain, mantel Cox log‐rank test analysis, **p < 0.01. (C) Gradient virulence test, 7‐week‐old ICR mice, 10 in each group, were infected with 10^2^ ME49 and 10^2^, 10^3^, 10^4^, 10^5^ ME49*Δ203240* strain, mantel Cox log‐rank test analysis, **p < 0.01. (D) Brain cyst loads. The surviving mice in (B) were sacrificed, and the number of brain cysts were stained with DBA‐FITC and counted under a fluorescence microscope, *p < 0.05, unpaired *t*‐test. (E) DBA staining of brain cysts. Tissue cysts stained with DBA‐FITC and imaged on fluorescence microscopy. Scale bar = 5 μm. (F) HE stained liver, lung and spleen sections from mice injected with ME49*Δ203240* and ME49 strains after 30 days. Arrows represent pathological changes. In mice infected with the ME49 strain, the arrows in the histopathological sections of the liver represent foci of mixed cell infiltration (macrophages and neutrophils), the arrows in the lungs represent a large perivascular mixed cell infiltration (macrophages and neutrophils), and the arrows in the spleen represent a severe reduction in the number of lymphocytes within the splenic white and red marrow.

To evaluate whether the deletion of the TGME49_203240 gene impacts the replication and proliferation rate of tachyzoites in vivo, fluorescence qPCR was used to quantify the relative content of tachyzoites in the peritoneal fluid of mice. The results showed that the proliferation rate of the ME49*Δ203240* strain was reduced significantly in mice compared with the wild type (Figure 4A). These findings suggest that TGME49_203240 gene deletion leads to a significant reduction in the replication and proliferation rate of tachyzoites in vivo.

### 3.5 TGME49_203240 Deletion Results in Reduced Parasite Virulence and Reduced Tissue Cyst Formation

Virulence experiments and gradient virulence experiments were conducted to investigate whether the deletion of the TGME49_203240 gene affects the virulence of *T*. *gondii*. In the virulence experiment, the 30‐day survival curves of mice are shown in Figure 4B, and the survival rate of mice injected with the ME49*Δ203240* strain was 100%, whereas the mortality rate of mice injected with the wild‐type ME49 strain was 70%. The result suggests that the deletion of the TGME49_203240 gene reduces the virulence of parasites. Subsequently, to further confirm the extent of reduced virulence of *Toxoplasma* after deletion of the TGME49_203240 gene, the gradient virulence assay was performed, and mice were injected intraperitoneally with 10^2^, 10^3^, 10^4^ and 10^5^ ME49*Δ203240* strains, while the controls were injected with 200 ME49 strains. As shown in Figure 4C, inoculation with 10^4^ and 10^5^ ME49*Δ 203240* strains resulted in the death of all mice within 30 days, with a survival rate of 30% in the 10^3^ group, 80% in the 10^2^ group and 10% in the mice inoculated with wild‐type ME49 strains, suggesting that the deletion of the TGME49_203240 gene reduced the virulence of *T*. *gondii* to a certain degree, but the ME49*Δ203240* strain remained highly virulent at high doses.

In addition, the ability to form brain cysts of the ME49*Δ203240* strain was evaluated. After being injected with 200 tachyzoites of the ME49*Δ203240* strain for 30 days, live mice were euthanised, and their brain tissues were collected for counting. The results are shown in Figure 4D,E. Compared with mice injected with the ME49 strain, those mice injected with the ME49*Δ203240* strain had a significantly lower number of brain cysts (*p* < 0.05). This indicates that deletion of the TGME49_203240 gene significantly reduces the ability of *T. gondii* to form brain cysts.

To further evaluate the safety of the ME49*Δ203240* strain, lung, spleen and liver tissues were collected from mice injected with 200 ME49*Δ203240* and ME49 tachyzoites after 30 days and examined for tissue damage by HE staining. Histopathological analysis revealed almost no significant changes in the tissue sections of mice immunised with ME49*Δ203240* strain compared to the uninfected mice (Figure 4F). In contrast, mice infected with wild‐type ME49 exhibited marked inflammation in the liver and lungs, characterised by macrophage and neutrophil infiltration. Lung tissue displayed alveolar wall thickening and structural damage, while the spleen showed a severe reduction in lymphocytes in both the white and red pulp.

### 3.6 The Complement of TGME49_203240 Restores *T. gondii* In Vitro Growth Defects and In Vivo Virulence

**FIGURE 5:**
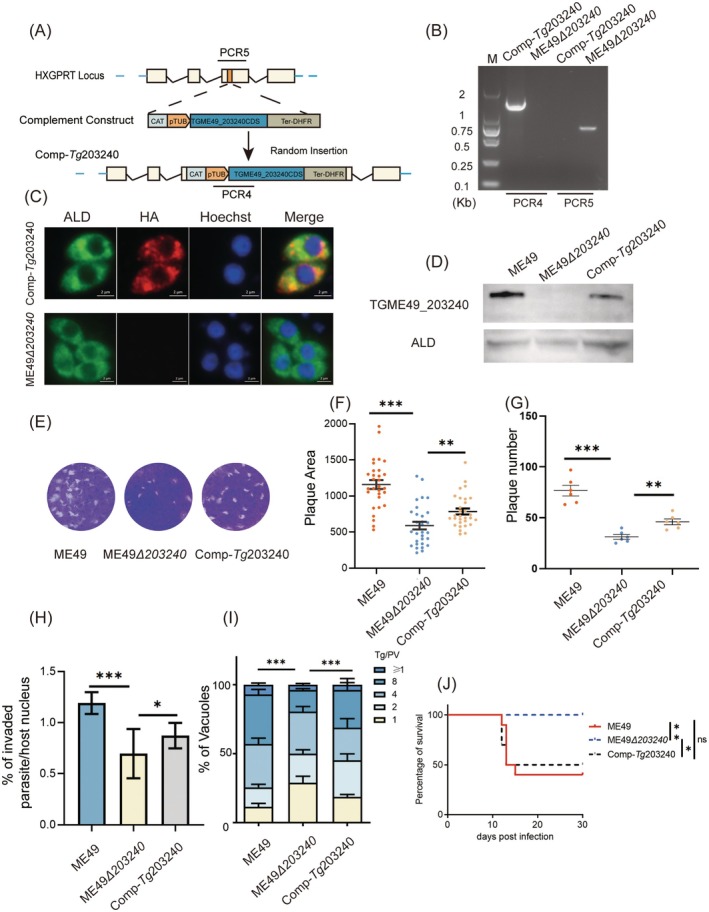
The complement of TGME49_203240 restores *T. gondii* in vitro growth defects and in vivo virulence. (A) Schematic diagram of Comp‐*Tg*203240 strain construction. (B) PCR identification of Comp‐*Tg*203240 strain. PCR4 represents the successful insertion of the TGME49_203240 CDS sequence, and PCR5 represents the disruption of the HXGPRT site. (C) IFA identification of Comp‐*Tg*203240 complementation strain. Scale bar = 2 μm. (D) Western Blot identification of Comp‐*Tg*203240 strain with complement of TGME49_203240. Primary antibodies were selected from mouse‐anti‐TGME49_203240 and rabbit‐anti‐*Tg*ALD. (E–I) ME49, ME49*Δ203240* and Comp‐*Tg*203240 phenotyping experiments. (E) Plaque assay, 200 parasites infected HFF cells, grown for 10 days and stained with crystal violet. (F) Plaque area, unpaired *t*‐test, ***p < 0.001, **p < 0.01. (G) Plaque number, unpaired *t*‐test, ***p < 0.001, **p < 0.01. (H) Invasive capability assay, ***p < 0.001, *p < 0.05, two‐way ANOVA. (I) Replication assay, ***p < 0.001, two‐way ANOVA. (J) Virulence test, seven‐week‐old ICR mice, 10 in each group, were infected with 200 ME49, ME49*Δ203240* and Comp‐*Tg*203240 strain, **p < 0.01, *p < 0.05, ns, not significant, mantel Cox log‐rank test analysis.

To verify that the reduced virulence and growth defects observed in ME49*Δ203240* strain were indeed due to the deletion of the TGME49_203240 gene, a complement strain (Comp‐*Tg*203240) was constructed by inserting the CDS fragment of TGME49_203240 into the HXGPRT locus of the ME49*Δ203240* strain (Figure 5A). Following the selection with chloramphenicol, PCR analysis confirmed the correct insertion of the TGME49_203240 CDS fragment (Figure 5B). IFA further verified the successful construction of the Comp‐Tg203240 strain by detecting the expression of the HA‐tagged protein (Figure 5C). Additionally, Western blot analysis demonstrated the presence of TGME49_203240 in the Comp‐Tg203240 strain, while no expression was detected in ME49Δ203240, confirming the successful restoration of TGME49_203240 expression (Figure 5D).

Plaque assay results demonstrated that the Comp‐*Tg*203240 strain formed larger plaques compared to the ME49*Δ203240* strain (Figure 5E), with a statistically significant difference in plaque area and numbers between the two strains (Figure 5F,G). Furthermore, the results of the invasive capability and replication assay showed that the phenotypic defects of the ME49*Δ203240* strain were somewhat restored after complement with TGME49_203240 CDS (Figure 5H,I). These results further verified that TGME49_203240 is important for the in vitro growth of *T*. *gondii*.

The virulence experiments showed that the survival rate of mice infected with the wild‐type ME49 strain was 40%, mice infected with the Comp‐*Tg*203240 strain had a 50% survival rate while mice infected with the ME49*Δ203240* strain had a 100% survival rate (Figure 5J). These results suggest that the complementation of TGME49_203240 significantly enhances virulence, highlighting the critical role of TGME49_203240 in *T. gondii* virulence.

### 3.7 ME49*Δ203240* Vaccination Provides Robust Immune Protection in Mice

The deletion of the TGME49_203240 gene significantly reduced the virulence of *T*. *gondii* and its proliferative ability in mice, which made us speculate that the ME49*Δ203240* strain may be a potential live attenuated vaccine against *T*. *gondii*. To evaluate whether the ME49*Δ203240* strain could provide immunoprotection in mice, immunoprotection experiments were performed.

**FIGURE 6:**
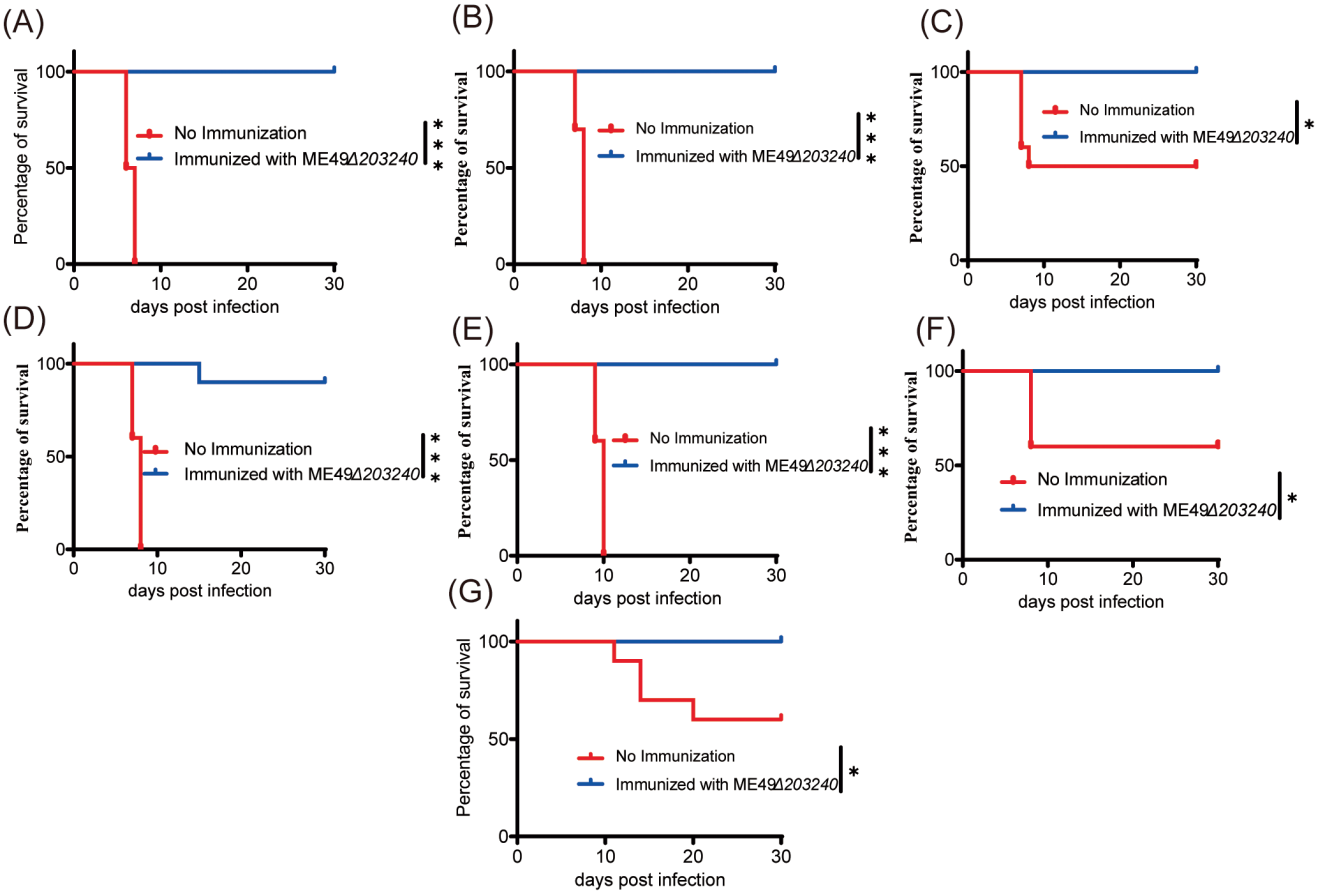
ME49*Δ203240* immunisation protected mice from *T. gondii* tachyzoite and cyst challenge. (A‐C) Seven‐week‐old ICR mice were immunised with ME49*Δ203240* tachyzoites. After 30 days post‐immunisation, the mice were challenged with 10^4^ tachyzoites of RH*ΔHXGPRT* (A), ME49 (B), or VEG (C) via intraperitoneal injection and their survival was monitored for another 30 days. (D–F) Seven‐week‐old ICR mice were immunised with ME49*Δ203240* tachyzoites. After 75 days post‐immunisation, the mice were challenged with 10^4^ tachyzoites of RH*ΔHXGPRT* (D), ME49 (E) or VEG (F) via intraperitoneal injection, and their survival was monitored for another 30 days. Unimmunised mice (*n* = 10 mice for each strain) were included as a control. (G) Seven‐week‐old ICR mice were immunised with ME49*Δ203240* tachyzoites. After 30 days post‐immunisation, the mice were challenged with 50 cysts of the ME49 strain, and the survival of the mice was monitored for 30 days. Unimmunised mice (*n* = 10 mice for each strain) were included as a control. Mantel‐Cox log‐rank test analysis, *p < 0.05, ***p < 0.001.

Following a 30‐day immunisation period with 200 ME49*Δ203240* strains, mice were challenged with 1 × 10^4^ *T. gondii* tachyzoites of type I strains (RH*ΔHXGPRT*), type II strains (ME49) and type III strains (VEG). The results demonstrated that the survival rate of all mice in the immunised group was 100%, whereas the survival rate of mice in the unimmunised group was 0% (type I), 0% (type II) and 50% (type III), respectively (Figure 6A–C). These findings suggest that the ME49*Δ203240* strain provides robust immune protection against the challenge of *T. gondii* I, II and III strains within 30 days. Moreover, to determine whether the ME49*Δ203240* strain could provide long‐term immune protection for mice, the duration of immunisation was extended to 75 days in this study. The results demonstrated that the survival rates of mice immunised with 200 ME49*Δ203240* strains for 75 days were 90% (type I), 100% (type II) and 100% (type III), whereas the survival rates of mice in the unimmunised group were 0% (type I), 0% (type II) and 60% (type III) (Figure 6D–F).

Besides, to evaluate whether ME49*Δ203240* vaccination could provide protective immunity against cyst infection, the mice immunised with ME49*Δ203240* were challenged with 50 cysts of ME49. The results showed that the survival rate of mice in the immunised group was 100%, whereas the survival rate of mice in the unimmunised group was 60% (Figure 6G). This indicates that ME49*Δ203240* vaccination effectively induced protective immunity against chronic *Toxoplasma* infection. In summary, these findings further indicated that the ME49*Δ203240* strain confers long‐term immunoprotection in mice following immunisation.

### 3.8 ME49*Δ203240* Vaccinated Mice Induced Strong Humoral and Cellular Immune Responses

To investigate the immunoprotective mechanisms induced by the ME49*Δ203240* strain against *T. gondii* challenge, we analysed specific antibody and cytokine responses in the sera of immunised mice.

**FIGURE 7:**
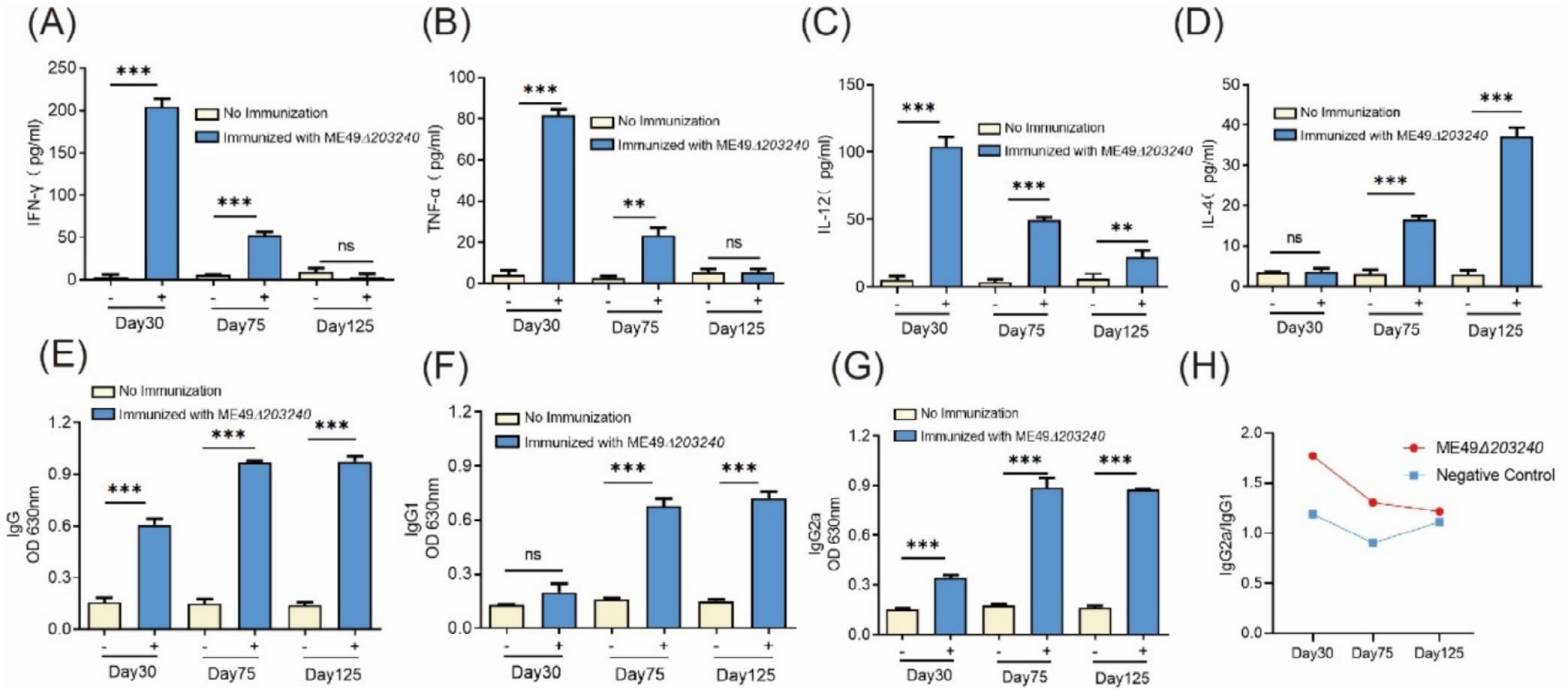
ME49*Δ203240* vaccinated mice induced strong humoral and cellular immune responses. (A–D) ELISA for detection of serum cytokines in mice, which immunised ME49*Δ203240* strain 30, 75 and 125 days. (A) IFN‐γ; (B) TFN‐α; (C) IL‐12; (D) IL‐4, ns = not significant, **p < 0.01, ***p < 0.001, unpaired *t*‐test. (E and F) ELISA for detection of serum‐specific antibodies in mice, which immunised ME49*Δ203240* strain 30 days, 75 days and 125 days. (E) IgG; (F) IgG1; (G) IgG2a; (H) IgG2a/IgG1, yellow for unimmunised group, blue for immunised group, ns = not significant, ***p < 0.001, unpaired *t*‐test.

The results showed that the immunised mice produced high levels of IFN‐γ, TNF‐α and IL‐12 at 30 and 75 days post‐immunisation. However, after 75 and 125 days, IFN‐γ, TNF‐α and IL‐12 significantly declined compared to 30 days, suggesting that the immune response peaked early around day 30 and gradually waned over time. In contrast, IL‐4 was significantly elevated after days 75 and 125, indicating a potential activation of anti‐inflammatory mechanisms and a shift in the immune response over time (Figure 7A–D).

The results also showed that the level of specific IgG antibodies in mice inoculated with the ME49*Δ203240* strain gradually increased with time, peaking at about 75 days and maintaining a high level of IgG at 125 days. This suggests that the ME49*Δ203240* strain induced a strong humoral immune response in mice (Figure 7E). To further explore the link between the immune responses immunised in mice by the ME49*Δ203240* strain, we examined the IgG2a and IgG1 antibodies. Based on the results of the IgG2a/IgG1 ratio, we found that after 30 days of immunisation of mice with the strain, the IgG2a/IgG1 ratio was much greater than 1 and the negative control, which indicated that the ME49*Δ203240* strain induced a Th1‐biased immune response in mice; however, the IgG2a/IgG1 ratio gradually decreased at 75 and 125 days, which may reflect the body's regulatory adjustment of the Th1/Th2 immune balance over time (Figure 7F–H).

In conclusion, immunisation with the ME49*Δ203240* strain induced a strong humoral immune response along with a Th1‐biased cellular immune response in mice, which in turn protected mice against the challenge of *T*. *gondii*.

## 4 Discussion

*T. gondii* is widespread globally, posing a significant threat to public health and farming. Nevertheless, there is currently no effective vaccine or drug available for the prevention and treatment of *T. gondii* infection (Chu and Quan 2021). In this study, we explored novel strategies for developing a *T. gondii* vaccine by targeting vesicular transport proteins which may play a critical role in the parasite's growth and virulence. Through database and bioinformatic analysis, we found a protein containing a C2 domain (TGME49_203240) and identified that it was localised in the cytoplasm and endoplasmic reticulum. Additionally, we evaluated the potential of the ME49*Δ203240* strain as a live attenuated vaccine candidate and investigated the immune protection mechanism it provides in mice. Our findings demonstrated that the ME49*Δ203240* strain conferred strong immune protection in mice.

Bioinformatics analysis suggests that TGME49_203240 may be involved in vesicular transport in *T*. *gondii*, a process essential for the parasite's life activities and virulence. Numerous proteins were found to be involved in the vesicular transport process of *T*. *gondii*, including members of the SNARE protein family, *T*. *gondii* SM proteins, endosomal vesicle plugging complex (HOPS), GTPase (DrpB) and Rab (Rab11a, Rab11b) proteins, *etc*. (Breinich et al. 2009; Agop‐Nersesian et al. 2010; Jackson et al. 2013; Cao et al. 2020; Shu et al. 2020; Venugopal et al. 2020; Fu et al. 2023). Additionally, proteins containing the C2 domain, such as Ferlin family proteins and *Tg*DOC2, have also been implicated in *T. gondii* vesicle translocation (Tagoe et al. 2021). It is reported that the C2 domain‐containing proteins may function as Ca^2+^‐dependent targeting proteins involved in membrane translocation and signal transduction (Nalefski and Falke 1996; Corbalan‐Garcia and Gomez‐Fernandez 2014).

In this study, we identified TGME49_203240, a C2 domain‐containing protein, through the ToxoDB database. Sequence analysis revealed that TGME49_203240 shares structural similarities with SNARE family proteins involved in vesicle fusion, with one side anchoring to the membrane and the other binding to interaction partners. Further sequence comparison, we found that the C2 domain of TGME49_203240 resembles that of C2D_Tricalbin‐like proteins and has two Ca^2+^ binding sites. We also found that TGME49_203240 is localised in the cytoplasm and endoplasmic reticulum of *T. gondii*, which further suggests that TGME49_203240 may be involved in vesicular transport. However, TGME49_203240 does not colocalise with gliding‐associated protein (*Tg*GAP45), suggesting that TGME49_203240 may be involved in the transport of substances between the endoplasmic reticulum and other organelles as well as vesicle synthesis of *T*. *gondii* but not participating in extracellular vesicle transport. Additionally, deletion of TGME49_203240 had no significant effect on the localisation of cytoplasmic, Golgi, rhoptry, microneme, dense granule, or gliding‐associated proteins, indicating that its function may be distinct from known vesicular transport pathways and additional research may be needed to clarify the function of TGME49_203240 (Figure S3).

Researches support that some C2 domain‐containing proteins are essential for *T. gondii* growth. The absence of these proteins impacts *Toxoplasma* biological processes such as adhesion, invasion, proliferation, egress and tissue cyst formation. For example, the deletion of FER1 and FER2 of the Ferlin family of proteins affects protein secretion from *T. gondii* microsomes and rods (Coleman et al. 2018; Tagoe et al. 2021). Whereas knockdown of *Tg*DOC2, a protein containing a double C2 structural domain, affects the secretion of *Toxoplasma* microsomal proteins. The deletion of the RASP2 protein, a protein containing a C2 domain and a PH domain, affects the invasive ability of *Toxoplasma* tachyzoites and reduces rod‐shaped body protein secretion (Suarez et al. 2019). In our study, we found that TGME49_203240 gene deletion causes a defective phenotype characterised by reduced invasive and replicative capacity of *T. gondii*. Given that *T. gondii* FER1 and FER2 also have the same invasive ability‐deficient phenotype, we hypothesised that the functional impairment observed in the TGME49_203240 knockout strain may be attributed to its C2 domain, which is to be further investigated in subsequent studies.

The immunoprotective mechanisms against *T*. *gondii* are complex, involving both humoral and cellular immunity. As an intracellular parasite, *T. gondii* cannot be effectively controlled by high titers of specific IgG alone, making the cellular immune response crucial for protection (Chu and Quan 2021; Sana et al. 2022). Previous studies have shown that while many subunit vaccines for *T. gondii* elicit strong humoral immune responses, they often fail to provide adequate protection against highly virulent strains. For example, *Toxoplasma* tyrosine hydroxylase (*Tg*TH) provided only partial immunoprotection in mice, while subunit vaccines incorporating GRA2 and GRA5 enhanced the Th1‐type immune response in mice but only prolonged the survival of mice infected with RH strains of the parasite (Ching et al. 2016; Zhang et al. 2020). In contrast, live attenuated vaccines have demonstrated superior protective efficacy. For example, mice immunised with the ME49*Δ6gpdh* strain for 30 days exhibit a survival rate of 80% and 100% when challenged with *T. gondii* RH*Δku80* and ME49 strains, respectively (Guo et al. 2023). In this study, mice immunised with the ME49*Δ203240* strain for 30 days and subsequently challenged with *T. gondii* tachyzoites of RH*ΔHXGPRT*, ME49 and VEG strains, as well as cysts of the ME49 strain, exhibited a 100% survival. These findings indicate that the ME49*Δ203240* strain induced an immune response similar to that generated by natural *T. gondii* infections and provides robust protection against *T*. *gondii* challenge.

Meanwhile, we found that ME49*Δ203240* induced high levels of specific antibody IgG and cytokines such as cytokines IFN‐γ and TNF‐α compared to live attenuated vaccines such as *Δα‐amy*, *ΔADSL*, *etc*. (Wang et al. 2020; Yang et al. 2020). This suggests that the mechanism of immune response produced by the ME49*Δ203240* strain immunising the organism may be similar to that of these vaccines. To further characterise the humoral immune response, we examined the IgG antibody subtypes, IgG2a and IgG1, which are generally considered indicators of a Th1‐type and Th2‐type immune response, respectively. The IgG2a/IgG1 ratio, a commonly used marker for evaluating vaccine immune response, with a ratio greater than 1, indicating a Th1‐biased response, which is critical for the clearance of intracellular pathogens (Golkar et al. 2007; Xu et al. 2014; Abasi et al. 2019; Rostami et al. 2020). In this study, the IgG2a/IgG1 ratio of mice immunised with the ME49*Δ203240* strain at 30 days post‐immunisation was much greater than 1, indicating that the ME49*Δ203240* strain induced a Th1‐biased immune response in mice. These findings collectively demonstrated that ME49*Δ203240* is a highly promising attenuated *T*. *gondii* vaccine candidate. However, further evaluation of its immunoprotective efficacy in other host species is necessary, along with rigorous safety assessments. In addition, ME49*Δ203240* remains highly virulent at high doses and, like most live attenuated vaccines, can still form tissue cysts in mice. Therefore, co‐deletion with other genes could be considered to further reduce the virulence.

In conclusion, this study demonstrated that the deletion of TGME49_203240 impairs the ability of the *T. gondii strain* ME49 to invade, proliferate and form tissue cysts, resulting in a reduction of its virulence. Additionally, the immunoprotective efficacy and safety of the ME49*Δ203240* strain were evaluated in depth in this study. Our findings revealed that the ME49*Δ203240* strain induces a Th1‐biased cellular immune response as well as a humoral immune response in mice, providing immunoprotection against challenges with both *T. gondii* tachyzoites and bradyzoites. These results underscore the potential of TGME49_203240 as a promising target for the development of a live attenuated *T. gondii* vaccine. However, further refinement is necessary to ensure the safety and optimise the immunoprotection capabilities of the ME49*Δ203240* strain.

## Author Contributions

**Yifan Luo:** formal analysis, investigation, writing – original draft. **Mingfeng He:** investigation, methodology. **Shengqiang Yang:** investigation, methodology, writing – review and editing. **Jiahui Qian:** investigation, methodology, writing – review and editing. **Zhengming He:** investigation, methodology. **Jiayin Xu:** investigation, methodology. **Liyu Guo:** investigation, methodology. **Siyu Xiao:** conceptualization, data curation, formal analysis, funding acquisition, project administration, resources, supervision, visualization, writing – original draft, writing – review and editing. **Rui Fang:** conceptualization, data curation, formal analysis, funding acquisition, project administration, supervision, resources, writing – original draft, writing – review and editing, visualization.

## Ethics Statement

All 7‐week‐old female ICR mice were purchased from Hubei Provincial Laboratory Animal Research Centre, Licence No.: SCXK‐2020‐0084. All mice were kept in a standard SPF environment according to the requirements of the Animal Ethics Committee of Huazhong Agricultural University, following the requirements of animal welfare and related ethical requirements (Approval No.: HZAUMO‐2024‐0076).

## Conflicts of Interest

The authors declare no conflicts of interest.

## Supporting information


Figure S1.–S3.

## Data Availability

The data that support the findings of this study are available from the corresponding author upon reasonable request.
