# Supplementary material for: Toxoplasma gondii C2 Domain Protein Deletion Mutant as a Promising Vaccine Against Toxoplasmosis in Mice
Source: Microb Biotechnol. 2025 May 23;18(5):e70143. doi: 10.1111/1751-7915.70143 (PMC12101070; doi:10.1111/1751-7915.70143)
Supplement: Supplementary file 1 — Figure S1.–S3. [file MBT2-18-e70143-s001.docx]

**Supplementary chart**

**Fig.S1**


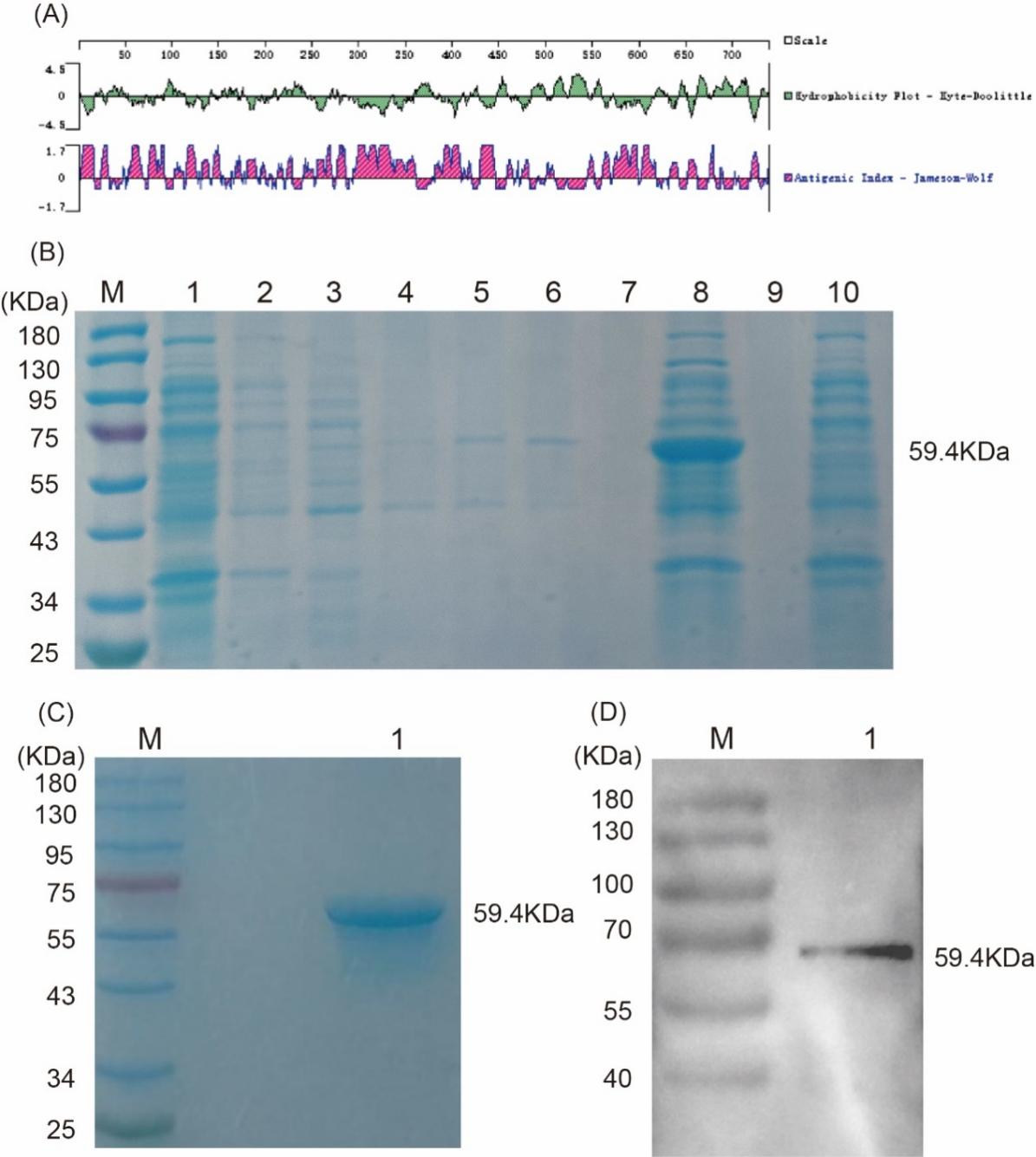


**Fig.S1** After constructing pET32a-TGME49_203240, the protein was expressed in *E. coli* BL21 (DE3), and a mouse-derived polyclonal antibody was prepared. (A) TGME49_203240 Hydrophobicity Flot and Antigenic Index analysis. After analyzing TGME49_203240 by DNASTARS, amino acids 1-414 were selected for protein expression. (B) TGME49_203240 protein expression. After inducing protein expression, *E. coli* was crushed, and a nickel column was used for protein binding and eluted with an imidazole solution. M: marker. 1: effluent. 2: heterogenous wash. 3: 40mM imidazolium eluent. 4: 80mM imidazolium eluent. 5: 120mM imidazolium eluent. 6: 400mM imidazolium eluent. 8: inclusion body. 10: supernatant. (C) SDS-PAGE identification of inclusion body purified protein. M: marker. 1: Inclusion body purified protein. (D) Western Blot to identify the binding ability of polyclonal antibody to antigen. The expressed TGME49_203240 protein was added to SDS to make samples and incubated with the prepared TGME49_203240 mouse-derived polyclonal antibody as the primary antibody and HRP-goat anti-mouse as the secondary antibody.

**Fig.S2**


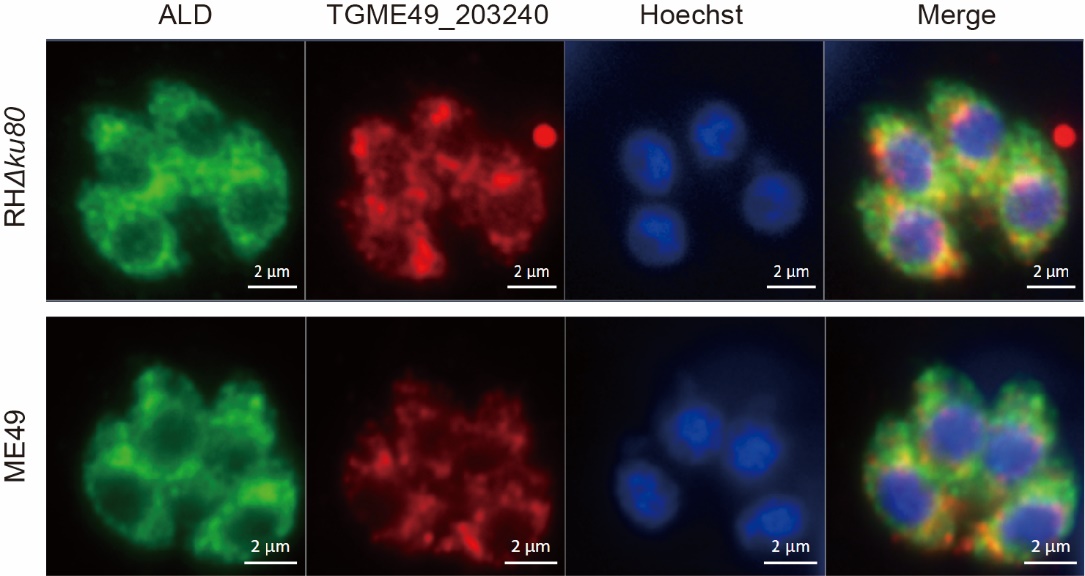


**Fig.S2** IFA assay for subcellular localization of the 203240 gene expression protein in *T. gondii* RH and ME49 strains. Scale bar=2μm. IFA assay was performed using mouse anti-TGME49_203240 and rabbit anti-*Tg*ALD antibodies as primary antibody.

**Fig.S3**


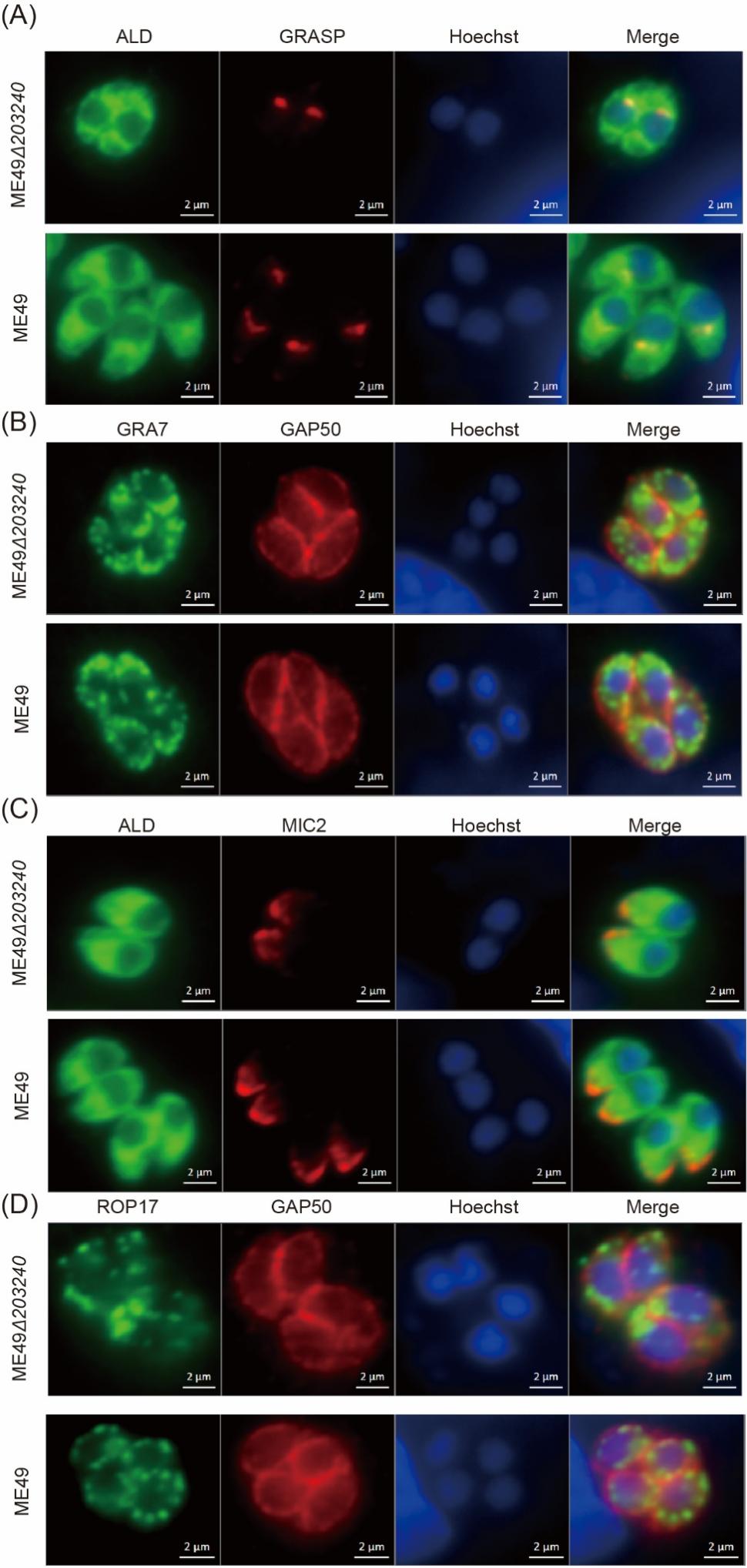


**Fig.S3** IFA detection of the effect of TGME49_203240 gene deletion on individual organelle marker proteins(ALD、GRASP、GRA7、GAP50、MIC2、ROP17). ALD represents cytoplasmic protein; GRASP represents Golgi protein; GRA7 represents *T. gondii* dense granule protein; GAP50 represents gliding-associated protein; MIC2 represents microneme protein; ROP17 represents rhoptry protein. Scale bar=2μm. TGME49_203240 gene deletion does not affect the localization of individual organelle protein markers
